# Supplementary material for: A framework to measure the taxonomic of economic anchor: A case study of the Three Seas Initiative countries
Source: PLoS One. 2021 May 27;16(5):e0252292. doi: 10.1371/journal.pone.0252292 (PMC8158954; doi:10.1371/journal.pone.0252292)
Supplement: S1 Table — (DOCX) [file pone.0252292.s001.docx]

| **Country** | **Value added per employee** | **Share of employees in agriculture. forestry and fishing** | **Share of employees in manufacturing** | **Share of employees in trade** | **Share of persons working in accommodation and food service activities** | **Population density** | **Share of unemployed in the total numer of active people** | **Share of persons of working age (15-64)** | **Export of goods and services (% in GDP)** | **Imports of goods and services (% of GDP)** | **Innovation index** | **Use of accommodation capacity** | **Tourist traffic intensity-Schneider’s index** | **Tourist traffic intensity-Charvata’s index** |
| --- | --- | --- | --- | --- | --- | --- | --- | --- | --- | --- | --- | --- | --- | --- |
| **2015** | | | | | | | | | | | | | | |
| Austria | 73072.62 | 3.97 | 16.21 | 14.49 | 5.72 | 102.27 | 5.70 | 67.27 | 82.66 | 75.16 | 0.56 | 347.82 | 343.49 | 1016.85 |
| Bulgaria | 12523.76 | 6.74 | 19.84 | 17.16 | 5.26 | 64.89 | 9.20 | 66.14 | 77.81 | 76.39 | 0.22 | 112.23 | 79.75 | 272.90 |
| Croatia | 23218.46 | 8.26 | 16.86 | 14.36 | 6.52 | 74.73 | 16.20 | 66.48 | 46.85 | 39.29 | 0.25 | 138.72 | 146.66 | 524.37 |
| Czechia | 28693.41 | 2.91 | 27.61 | 12.26 | 3.93 | 133.61 | 5.10 | 66.96 | 64.54 | 63.57 | 0.41 | 172.46 | 130.57 | 325.10 |
| Estonia | 28729.73 | 3.85 | 19.13 | 13.28 | 4.08 | 29.00 | 6.20 | 65.30 | 81.05 | 75.05 | 0.43 | 34.12 | 201.21 | 361.01 |
| Hungary | 21399.95 | 4.85 | 21.50 | 12.85 | 4.47 | 105.96 | 6.80 | 67.62 | 68.82 | 69.82 | 0.31 | 77.75 | 90.18 | 211.60 |
| Latvia | 24001.84 | 7.73 | 13.10 | 14.51 | 3.48 | 30.31 | 9.90 | 65.62 | 121.97 | 93.17 | 0.29 | 19.23 | 93.92 | 169.88 |
| Lithuania | 25406.89 | 8.83 | 15.40 | 17.05 | 2.59 | 44.66 | 9.10 | 66.71 | 29.72 | 26.70 | 0.31 | 21.16 | 67.07 | 123.69 |
| Poland | 23060.08 | 11.34 | 19.46 | 14.56 | 2.13 | 121.09 | 7.50 | 69.55 | 49.50 | 46.40 | 0.25 | 46.56 | 52.98 | 102.10 |
| Romania | 16172.43 | 23.08 | 18.81 | 13.92 | 2.26 | 83.12 | 6.80 | 67.51 | 41.02 | 41.64 | 0.14 | 46.37 | 40.75 | 99.37 |
| Slovakia | 28623.84 | 3.16 | 24.81 | 12.29 | 4.72 | 110.58 | 11.50 | 70.73 | 77.15 | 69.15 | 0.32 | 82.95 | 62.33 | 154.23 |
| Slovenia | 36082.52 | 5.83 | 23.72 | 12.30 | 5.14 | 101.73 | 9.00 | 67.34 | 92.02 | 88.95 | 0.46 | 110.19 | 126.30 | 336.37 |
| **2016** | | | | | | | | | | | | | | |
| Austria | 74115.36 | 3.85 | 16.15 | 14.43 | 6.12 | 103.65 | 6.00 | 67.22 | 79.54 | 69.32 | 0.59 | 347.25 | 353.60 | 1035.40 |
| Bulgaria | 13347.90 | 6.65 | 19.67 | 17.16 | 5.27 | 64.45 | 7.60 | 65.61 | 79.38 | 78.18 | 0.23 | 110.84 | 92.15 | 325.97 |
| Croatia | 23597.86 | 6.79 | 17.23 | 13.94 | 6.89 | 74.12 | 13.10 | 66.20 | 46.02 | 38.66 | 0.25 | 141.43 | 154.46 | 555.11 |
| Czechia | 30224.49 | 2.87 | 28.16 | 11.84 | 3.55 | 133.81 | 4.00 | 66.30 | 64.73 | 59.72 | 0.40 | 173.81 | 140.96 | 345.78 |
| Estonia | 29465.13 | 3.87 | 19.11 | 13.13 | 4.21 | 29.02 | 6.80 | 64.91 | 79.56 | 71.84 | 0.39 | 34.26 | 214.29 | 384.06 |
| Hungary | 21901.45 | 4.99 | 21.80 | 12.49 | 4.55 | 105.69 | 5.10 | 67.23 | 67.58 | 66.80 | 0.32 | 79.33 | 96.75 | 231.31 |
| Latvia | 24895.28 | 7.60 | 14.00 | 14.02 | 3.50 | 30.05 | 9.60 | 65.12 | 120.82 | 105.23 | 0.28 | 19.55 | 99.86 | 180.18 |
| Lithuania | 25501.71 | 7.68 | 15.66 | 17.42 | 2.67 | 44.16 | 7.90 | 66.34 | 29.33 | 26.04 | 0.37 | 21.29 | 72.55 | 132.77 |
| Poland | 24005.77 | 10.41 | 20.40 | 14.60 | 2.34 | 120.97 | 6.20 | 69.00 | 52.19 | 48.16 | 0.26 | 50.25 | 59.57 | 116.74 |
| Romania | 17257.70 | 20.73 | 19.53 | 14.37 | 2.51 | 82.66 | 5.90 | 67.10 | 41.19 | 42.11 | 0.15 | 46.36 | 44.96 | 107.45 |
| Slovakia | 29039.45 | 2.87 | 24.61 | 12.50 | 4.59 | 110.68 | 9.70 | 70.22 | 77.97 | 69.42 | 0.33 | 81.32 | 72.13 | 177.11 |
| Slovenia | 37220.72 | 4.23 | 25.37 | 12.94 | 4.50 | 101.80 | 8.00 | 66.74 | 93.73 | 90.78 | 0.47 | 110.72 | 137.15 | 362.60 |
| **2017** | | | | | | | | | | | | | | |
| Austria | 76134.21 | 3.45 | 15.78 | 14.38 | 6.16 | 104.51 | 5.50 | 67.07 | 83.39 | 72.64 | 0.59 | 346.71 | 363.78 | 1044.27 |
| Bulgaria | 13622.89 | 6.82 | 19.25 | 17.31 | 5.44 | 63.98 | 6.20 | 65.18 | 82.31 | 80.92 | 0.23 | 111.19 | 96.21 | 338.94 |
| Croatia | 24100.69 | 6.39 | 17.21 | 14.37 | 7.35 | 73.48 | 11.20 | 65.87 | 47.40 | 40.30 | 0.26 | 141.47 | 169.06 | 590.64 |
| Czechia | 31122.52 | 2.78 | 28.23 | 11.55 | 3.30 | 134.12 | 2.90 | 65.63 | 68.15 | 63.76 | 0.42 | 174.10 | 153.87 | 374.24 |
| Estonia | 29878.52 | 3.44 | 19.23 | 13.28 | 4.00 | 29.01 | 5.80 | 64.42 | 79.73 | 72.20 | 0.40 | 35.74 | 226.33 | 400.34 |
| Hungary | 22306.58 | 4.98 | 22.51 | 12.43 | 4.38 | 105.34 | 4.20 | 66.82 | 73.61 | 71.22 | 0.33 | 79.27 | 103.05 | 248.03 |
| Latvia | 25371.04 | 6.90 | 13.59 | 14.90 | 3.41 | 29.76 | 8.70 | 64.54 | 121.04 | 98.96 | 0.29 | 19.91 | 111.86 | 197.19 |
| Lithuania | 26807.98 | 7.39 | 15.61 | 17.16 | 2.58 | 43.54 | 7.10 | 65.86 | 30.84 | 27.86 | 0.36 | 21.35 | 78.87 | 146.50 |
| Poland | 23434.06 | 10.01 | 21.02 | 14.26 | 2.36 | 120.99 | 4.90 | 68.36 | 54.35 | 50.17 | 0.27 | 51.78 | 63.56 | 124.14 |
| Romania | 18276.90 | 20.28 | 19.89 | 14.39 | 2.42 | 82.17 | 4.90 | 66.64 | 41.47 | 43.59 | 0.16 | 47.85 | 49.20 | 113.23 |
| Slovakia | 29154.83 | 2.69 | 24.80 | 11.64 | 4.19 | 110.87 | 8.10 | 69.55 | 83.17 | 74.31 | 0.32 | 81.77 | 77.08 | 186.15 |
| Slovenia | 37045.89 | 4.64 | 25.50 | 12.37 | 4.74 | 101.88 | 6.60 | 66.16 | 95.11 | 92.92 | 0.47 | 112.98 | 153.61 | 393.76 |
| **2018** | | | | | | | | | | | | | | |
| Austria | 77888.50 | 3.32 | 16.33 | 14.49 | 5.92 | 105.10 | 4.90 | 66.90 | 84.32 | 73.33 | 0.60 | 348.40 | 373.36 | 1064.25 |
| Bulgaria | 14722.90 | 6.41 | 19.22 | 17.18 | 5.36 | 63.52 | 5.20 | 64.73 | 82.58 | 82.74 | 0.23 | 111.85 | 101.06 | 351.06 |
| Croatia | 24874.74 | 5.57 | 17.48 | 13.76 | 7.25 | 72.61 | 8.50 | 65.42 | 47.42 | 41.25 | 0.29 | 143.66 | 182.14 | 617.21 |
| Czechia | 33426.73 | 2.76 | 27.93 | 11.68 | 3.52 | 134.52 | 2.20 | 65.03 | 66.94 | 64.31 | 0.43 | 179.34 | 161.43 | 386.24 |
| Estonia | 32717.39 | 3.21 | 18.85 | 13.09 | 4.32 | 29.09 | 5.40 | 64.10 | 78.39 | 72.00 | 0.50 | 35.47 | 227.63 | 403.93 |
| Hungary | 24098.69 | 4.76 | 22.60 | 12.24 | 4.11 | 105.13 | 3.70 | 66.52 | 75.62 | 73.68 | 0.33 | 80.59 | 109.52 | 259.71 |
| Latvia | 26780.26 | 6.97 | 13.07 | 15.64 | 3.64 | 29.52 | 7.40 | 64.12 | 122.33 | 89.18 | 0.32 | 19.81 | 123.48 | 217.98 |
| Lithuania | 28688.45 | 7.01 | 16.18 | 17.43 | 2.51 | 42.94 | 6.20 | 65.35 | 31.45 | 28.95 | 0.39 | 22.85 | 87.21 | 158.08 |
| Poland | 25438.02 | 9.40 | 21.21 | 13.97 | 2.48 | 121.00 | 3.90 | 67.65 | 55.59 | 52.15 | 0.29 | 54.37 | 67.24 | 132.79 |
| Romania | 20280.63 | 19.77 | 19.94 | 14.78 | 2.63 | 81.69 | 4.20 | 66.19 | 41.64 | 44.85 | 0.16 | 48.59 | 52.04 | 119.48 |
| Slovakia | 29915.96 | 2.26 | 24.72 | 12.19 | 4.32 | 111.03 | 6.50 | 68.87 | 85.38 | 77.10 | 0.33 | 85.10 | 79.39 | 192.11 |
| Slovenia | 38829.61 | 4.36 | 25.21 | 12.46 | 4.47 | 101.93 | 5.10 | 65.55 | 96.09 | 94.06 | 0.42 | 112.98 | 153.54 | 403.64 |
|  |  |  |  |  |  |  |  |  |  |  |  |  |  |  |
|  |  |  |  |  |  |  |  |  |  |  |  |  |  |  |
|  |  |  |  |  |  |  |  |  |  |  |  |  |  |  |
|  |  |  |  |  |  |  |  |  |  |  |  |  |  |  |
|  |  |  |  |  |  |  |  |  |  |  |  |  |  |  |
|  |  |  |  |  |  |  |  |  |  |  |  |  |  |  |
| **2019** | | | | | | | | | | | | | | |
| Austria | 80523.99 | 3.32 | 16.06 | 14.42 | 6.13 | 105.53 | 4.50 | 66.73 | 84.32 | 73.33 | 0.60 | 346.51 | 381.62 | 1075.41 |
| Bulgaria | 15506.84 | 6.49 | 18.99 | 16.78 | 5.69 | 63.07 | 4.20 | 64.31 | 82.58 | 82.74 | 0.23 | 111.85 | 101.78 | 356.04 |
| Croatia | 25732.18 | 5.56 | 18.00 | 13.88 | 6.41 | 72.10 | 6.60 | 65.01 | 47.42 | 41.25 | 0.29 | 143.66 | 183.45 | 635.51 |
| Czechia | 36250.79 | 2.62 | 27.82 | 11.30 | 3.71 | 135.02 | 2.00 | 64.51 | 66.94 | 64.31 | 0.43 | 179.34 | 160.83 | 394.46 |
| Estonia | 35738.68 | 3.14 | 18.29 | 12.99 | 4.45 | 29.22 | 4.40 | 63.82 | 78.39 | 72.00 | 0.50 | 35.47 | 226.65 | 418.25 |
| Hungary | 25453.94 | 4.66 | 22.26 | 12.59 | 4.23 | 105.07 | 3.40 | 66.11 | 75.62 | 73.68 | 0.33 | 80.59 | 109.58 | 262.21 |
| Latvia | 28973.57 | 7.34 | 12.90 | 15.37 | 3.56 | 29.30 | 6.30 | 63.85 | 122.33 | 89.18 | 0.32 | 19.81 | 124.41 | 224.90 |
| Lithuania | 30716.83 | 6.24 | 16.11 | 16.98 | 2.86 | 42.72 | 6.30 | 65.13 | 31.45 | 28.95 | 0.39 | 22.85 | 87.67 | 172.44 |
| Poland | 27068.03 | 8.99 | 20.79 | 14.08 | 2.47 | 120.99 | 3.30 | 66.98 | 55.59 | 52.15 | 0.29 | 54.37 | 67.24 | 141.46 |
| Romania | 21997.42 | 19.06 | 19.45 | 15.06 | 2.64 | 81.21 | 3.90 | 65.81 | 41.64 | 44.85 | 0.16 | 48.59 | 52.35 | 125.31 |
| Slovakia | 31643.56 | 2.78 | 24.87 | 11.90 | 4.20 | 111.17 | 5.80 | 68.22 | 85.38 | 77.10 | 0.33 | 85.10 | 79.29 | 217.30 |
| Slovenia | 41083.32 | 3.69 | 25.86 | 12.88 | 4.12 | 102.62 | 4.50 | 65.07 | 96.09 | 94.06 | 0.42 | 112.98 | 152.50 | 397.09 |
