# Supplementary material for: A framework to measure the taxonomic of economic anchor: A case study of the Three Seas Initiative countries
Source: PLoS One. 2021 May 27;16(5):e0252292. doi: 10.1371/journal.pone.0252292 (PMC8158954; doi:10.1371/journal.pone.0252292)
Supplement: Attachment — Submitted filename: Response to Reviewer-MK-K_JU.docx [file pone.0252292.s002.docx]

Manuscript PONE-D-21-02343

Dear Carlos Alberto Zúniga-González, Ph.D.

Academic Editor PLOS ONE

Thank you for giving us the opportunity to submit a revised draft of the manuscript “A framework to measure the taxonomic of economic anchor: a case study of the Three Seas Initiative countries” for publication in the PLOS ONE.

We appreciate the time and effort that you and the reviewers dedicated to providing feedback on our manuscript and are grateful for the insightful comments on and valuable improvements to our paper. We have incorporated all the suggestions made by the reviewers. Those changes are highlighted within the manuscript.

Please find our detailed responses below:

Reviewer #1:

1: *The paper is interesting. However, I believe there are several indexes available that partially measures the entrepreneurial potential of countries. For instance, the world bank has "Ease of doing business index". In fact, the doing business project of the World Bank incorporates many of the indicators that might be helpful for the discussion.*

*The authors may wish to discuss how their measure differs from the other alternative measures, and why their measure is important.*

**Author response: Thank you for your suggestion on this very essential point.**

We agree with the above comment that there are several indicators available that partially measure the entrepreneurial potential of countries. These include those listed in the annual Doing Business report - the flagship publication of the World Bank Group. Doing Business includes 11 indicators to measure aspects of business regulation that are relevant to entrepreneurship. We used 4 of these in our work (they are included in Table 7).

In designing our economic anchor indicator, we aimed to create an indicator that reflects as fully as possible the factors that influence the development of entrepreneurship. To this end, 14 diagnostic features were selected, of which, after preliminary statistical analysis, 9 were used. In our opinion, the selected 9 features constitute the necessary basis for entrepreneurship development.

In addition, a relative pattern method based on Weber's median was used to develop an innovative economic anchor index. The advantage of this indicator is the easy interpretation of the obtained results, since the values of the relative measure Φ_it^((2)) can be greater or less than 1, which makes it possible to determine the relative position of an object, in this case countries, in relation to all others (lines 262-267).

2: *The paper mentions that the measure developed in the study can help improve "the ability to overcome the consequences of extraordinary events, such as COVID-19 and prospects for the return to an accelerated development once the destabiliser of the economic system", However, I think the discussion section of the paper, although focuses on Covid-19, is only weakly correlated with the results of the paper. I think the authors ought to strengthen the discussion section, and further the linkage between the analysis conducted in the paper and Covid-19*.

**Author response: Thank you for your kindly suggestion.**

Due to the lack of available data on the economic situation of individual countries in the period under review, it was not possible to deepen the analysis during the preparation of the text. Nevertheless, in accordance with the reviewer's suggestion, the discussion part of the article was strengthened in an attempt to clarify the relationships. (line 507 to 541).

Reviewer #2

1: *The authors have selected an important dimension and presented the paper in relatively easy to read, for a general reader, manner. However, the study cannot be linked to COVID-19. COVID-19 was first case was registered in China in the last quarter of 2019. In the selected countries it spread afterwards. So, the impact of COVID cannot be determined. For this, additional data of 2020 is required and it could be a much better before after situation analyses*.

**Author response: Thank you for your kindly suggestion.**

The reviewer's observation is correct. The first official case of COVID-19 in Europe was reported on 24 January 2020. The virus, which causes severe infectious disease, spread rapidly across the continent, and on 11 March the World Health Organisation declared a global pandemic. We wrote the article in July-October 2020, without yet having data on the changes in the economy caused by the pandemic. Being aware of this, we want to continue our research in the future. To articulate this in the paper we have added a paragraph on the limitations of our research (at the time we conducted it) and information on our future intentions (line 587 to 591).

Sincerely,

Magdalena Kozera-Kowalska (corresponding author)

Jarosław Uglis, Jarosław Lira
